# Supplementary material for: Neural signatures of auditory hypersensitivity following acoustic trauma
Source: eLife. 2022 Sep 16;11:e80015. doi: 10.7554/eLife.80015 (PMC9555866; doi:10.7554/eLife.80015)
Supplement: Supplementary file 1. [file elife-80015-supp1.docx]

|  | **Go/No Go Tone Detection**  **(N = 13, 7/6 Trauma/**  **Sham)** | **Go/No Go Tone and Optogenetic Detection**  **(N = 9, 6/3 Trauma/**  **Sham)** | **Chronic Widefield Imaging**  **(N = 2, 1/1 Trauma/**  **Sham)** | **Chronic Two-Photon Imaging**  **(N = 8, 4/4 Trauma/**  **Sham)** | **Chronic cochlear function testing**  **(N = 12 Trauma)** |
| --- | --- | --- | --- | --- | --- |
| **Headplate or Implantation Surgery** | Day -21 | Day -21 | Day -11 | Day -11 | -- |
| **Pre-exposure ABR/DPOAE** | D-20 | D-20 | D-7 | D-7 | Day -7 |
| **Behavioral Training** | D-19 to D-5 | D-19 to D-5 | -- | -- | -- |
| **Widefield Mapping** | -- | -- | D-5 | D-5 | -- |
| **Baseline Imaging** | -- | -- | D-4 to D-1 | D-4 to D-1 | -- |
| **Baseline Behavior** | D-4 to D-1 | D-4 to D-1 | -- | -- | -- |
| **Sham/Noise Exposure (all at age of 9 weeks postnatal)** | Day 0 | Day 0 | Day 0 | Day 0 | Day 0 |
| **Post-exposure Imaging** | -- | -- | D0 to D30 | D0 to D21 | -- |
| **Post-exposure Behavior** | D0 to D21 | D0 to D10 | -- | -- | -- |
| **Post-exposure ABR/DPOAE/Histology** | D21 | D21 | D30 | D21 | D0 to D21 |
